# Supplementary material for: An exploratory review of resiliency assessments after brain injury
Source: PLoS One. 2025 Jan 3;20(1):e0292502. doi: 10.1371/journal.pone.0292502 (PMC11698413; doi:10.1371/journal.pone.0292502)
Supplement: S1 Table — (DOCX) [file pone.0292502.s001.docx]

**S1 Appendix. Table 1. Search Terms**

| Database | | Concept 1  Resilience | Concept 2  Brain Injury | Concept 3  Clinical Measure | Concept 4  Psychometric Properties |
| --- | --- | --- | --- | --- | --- |
| CINAHL | Keywords | “resilienc*” or “resilient” or “self-efficacy” or “self-confidence” or “self-acceptance” or “self-worth” or “self-concept” or “emotional adjustment” | brain injur* or traumatic brain injur* or stroke or cerebrovascular accident* or cerebral vascular accident* | patient reported outcome or prom or self report or outcome assessment or measure* or assessment or questionnaire or rating scale | reliab* or valid* or clinical utility or psychometric* or psychometric properties |
|  | Subject headings | (MH "Hardiness") OR (MH "Confidence") OR (MH "Self Concept+") OR (MH "Confidence") OR (MH "Self-Compassion") OR (MH "Self-Efficacy") | (MH "Brain Injuries") OR (MH "Left Hemisphere Injuries") OR (MH "Right Hemisphere Injuries") OR (MH "Stroke+") | (MH "Patient-Reported Outcomes") OR (MH "Self Report") OR (MH "Outcome Assessment") | (MH "Reliability and Validity+") OR (MH "Psychometrics”) |
| MEDLINE* | Keywords | resilienc* or resilient or self-efficacy or self-confidence or self-acceptance or self-worth or self-concept or emotional adjustment | brain injur* or traumatic brain injur* or stroke or cerebrovascular accident* or cerebral vascular accident* | patient reported outcome or prom or self report or outcome assessment or measure* or assessment or questionnaire or rating scale | reliab* or valid* or clinical utility or psychometric* or psychometric properties |
|  | Subject headings | Resilience, Psychological/ or self-concept/ or self-efficacy/ | Brain Injuries, Traumatic/ or exp Stroke/ or Stroke Rehabilitation/ or Brain Injuries/ | Patient reported outcome measures/ or self-report/ or Outcome Assessment, Healthcare/ | “reproducibility of results”/ or Social Validity, Research/ or Psychometrics/ |
| PsycINFO | Keywords | resilienc* or resilient or self-efficacy or self-confidence or self-acceptance or self-worth or self-concept or emotional adjustment | brain injur* or traumatic brain injur* or stroke or cerebrovascular accident* or cerebral vascular accident* | patient reported outcome or prom or self report or outcome assessment or measure* or assessment or questionnaire or rating scale | reliab* or valid* or clinical utility or psychometric* or psychometric properties |
|  | Subject headings | DE "Resilience (Psychological)" OR DE "Self-Concept" OR DE "Self-Confidence" OR DE "Self-Esteem" OR DE "Self-Acceptance" | "Neurorehabilitation" OR DE "Neuropsychological Rehabilitation" OR  DE "Traumatic Brain Injury" OR DE "Brain Injuries" OR DE "Head Injuries" | DE "Patient Reported Outcome Measures" OR DE "Self-Report" | DE "Psychometrics" OR DE "Classical Test Theory" OR DE "Consistency (Measurement)" OR DE "Error of Measurement" OR DE "External Validity" OR DE "Factor Analysis" OR DE "Internal Validity" OR DE "Item Analysis (Test)" OR DE "Item Response Theory" OR DE "Measurement Invariance" OR DE "Measurement Models" OR DE "Multivariate Analysis" OR DE "Test Construction" OR DE "Test Reliability" OR DE "Test Sensitivity" OR DE "Test Specificity" OR DE "Test Validity" OR DE "Variability Measurement"  DE "Test Reliability" OR DE "Internal Consistency" OR DE "Interrater Reliability" OR DE "Split-Half Reliability" OR DE "Test-Retest Reliability" OR DE "Psychometrics" OR DE "Classical Test Theory" OR DE "Consistency (Measurement)" OR DE "Error of Measurement" OR DE "External Validity" OR DE "Factor Analysis" OR DE "Internal Validity" OR DE "Item Analysis (Test)" OR DE "Item Response Theory" OR DE "Measurement Invariance" OR DE "Measurement Models" OR DE "Multivariate Analysis" OR DE "Test Construction" OR DE "Test Reliability" OR DE "Test Sensitivity" OR DE "Test Specificity" OR DE "Test Validity" OR DE "Variability Measurement" |
| EMBASE | Keywords | resilienc* or resilient or self-efficacy or self-confidence or self-acceptance or self-worth or self-concept or emotional adjustment | brain injur* or traumatic brain injur* or stroke or cerebrovascular accident* or cerebral vascular accident* | patient reported outcome or prom or self report or outcome assessment or measure* or assessment or questionnaire or rating scale | reliab* or valid* or clinical utility or psychometric* or psychometric properties |
|  | Subject Headings | Psychological resilience/ or social resilience/ or self concept/ or “sense of self”/ | Brain injury/ or acquired brain injury/ or exp traumatic brain injury/ or cerebrovascular accident/ | Patient-reported outcome/ or self report/ or outcome assessment/ | Exp reliability/ or exp validity/ |
