# Supplementary material for: An exploratory review of resiliency assessments after brain injury
Source: PLoS One. 2025 Jan 3;20(1):e0292502. doi: 10.1371/journal.pone.0292502 (PMC11698413; doi:10.1371/journal.pone.0292502)
Supplement: S4 Table — (DOCX) [file pone.0292502.s004.docx]

Supporting Information 3. Data Extracted from Primary Research Sources

| Article | Eligible (Y/N) | Extractor (author) | Date |
| --- | --- | --- | --- |
| Kortte et al., 2009 | Y | JG | April 2023 |
| Whiting, Diane L; Deane, Frank P; Ciarrochi, Joseph; McLeod, Hamish J; Simpson, Grahame K, 2015 | Y | JG | April 2023 |
| Horne, Jane C.; Lincoln, Nadina B.; Logan, Pip A., 2017 | Y | JG | April 2023 |
| Stoner et al., 2015 | Y | JG | April 2023 |
| Maujean, Annick; Davis, Penelope; Kendall, Elizabeth; Casey, Leanne; Loxton, Natalie, 2014 | Y | JG | April 2023 |
| Carlstedt, E, Eva Månsson Lexell, Hélène Pessah-Rasmussen, and Susanne Iwarsson, 2015 | Y | JG | April 2023 |
| Lee, Danbi; Fogg, Louis; Baum, Carolyn M.; Wolf, Timothy J.; Hammel, Joy, 2018 | Y | JG | April 2023 |
| Losoi, Heidi, Noah D. Silverberg, Minna Wäljas, Senni Turunen, Eija Rosti-Otajärvi, Mika Helminen, Teemu Miikka Artturi Luoto, Juhani Julkunen, Juha Öhman, and Grant L. Iverson. 2015 | Y | JG | April 2023 |
| Longworth, Catherine; Deakins, Joseph; Rose, David; Gracey, Fergus, 2018 | Y | JG | April 2023 |
| Partridge, Cecily; Reid, Fiona; Jones, Fiona, 2008 | Y | JG | April 2023 |
