# Supplementary material for: An exploratory review of resiliency assessments after brain injury
Source: PLoS One. 2025 Jan 3;20(1):e0292502. doi: 10.1371/journal.pone.0292502 (PMC11698413; doi:10.1371/journal.pone.0292502)
Supplement: Attachment — Submitted filename: Response to Reviewers 4.docx [file pone.0292502.s005.docx]

**Formatting Changes**

| Editor Feedback | Author Response |
| --- | --- |
| Please state what role the funders took in the study. If the funders had no role, please state: “The funders had no role in study design, data collection and analysis, decision to publish, or preparation of the manuscript.” If this statement is not correct you must amend it as needed. Please include this amended Role of Funder statement in your cover letter; we will change the online submission form on your behalf. | We have now included a statement in our cover letter to indicate that the funders had no role in the study design, data collection and analysis, decision to publish, or preparation of the manuscript. |
| Please include captions for your Supporting Information files at the end of your manuscript, and update any in-text citations to match accordingly. Please see our Supporting Information guidelines for more information: <http://journals.plos.org/plosone/s/supporting-information>. | We have now provided supporting information files at the end of the manuscript and updated in-text citations. |

**Responses to Reviewers**

**Responses to Reviewer #1:**

| Reviewer feedback | Author Response | Location in manuscript |
| --- | --- | --- |
| The abstract does not clearly lay out the objective/question of the systematic review and an overview of the methods that will be used. | We have changed the abstract according to reviewer feedback with clear background, objective, and overview of the methods. | Abstract:  **Background:** People with brain injury can have lower resiliency compared to the general public. Yet, resiliency can facilitate positive processes to negotiate adversity after brain injury. It is therefore important to measure resiliency after a brain injury. **Objective:** The review aimed (1) to identify self-report resiliency outcome measures for use with people after acquired brain injury, using the process-based Traumatic Brain Injury Resiliency Model as the guiding conceptual framework, and (2) to summarize the psychometric properties of the identified outcome measures and the utility of these measures in clinical rehabilitation. **Method:** The COSMIN guidelines for systematic reviews were followed to ensure appropriate reporting for each measure. Databases CINAHL, EMBASE, Medline, and PsychINFO were searched and independently reviewed by two people. |
| Attach the strategy search for each database. | A comprehensive search strategy for each database has been added to Supplemental Material 1. | Supplementary Material 1. Search Terms |
| Explain data management and data synthesis. | We have now included information on the data management and synthesis, in accordance with the PRISMA guidelines. | Page 9, paragraph 1:  “The search results from each of the four databases were merged into Covidence, a systematic review management platform. Title and abstract screening was completed by two independent reviewers (authors JG and JS). Conflicts were resolved with conversations. Full text review took place by two independent reviewers (JG and JS) with conflicts resolved through discussion.” |
| How is the risk of bias in studies checked? | We thank the reviewer for raising this comment. Our review focused on the quality and content of resiliency assessments themselves, as opposed to the quality of the studies from which the assessments were identified. We therefore chose to provide psychometric properties of each assessment to provide validity and reliability information on each assessment. |  |

**Responses to Reviewer #2:**

| Reviewer Feedback | Author Response | Location in Manuscript |
| --- | --- | --- |
| In the abstract, it is suggested to provide a more detailed explanation of the nine resilience measurements identified, along with a summary of their respective psychometric properties. | We thank the reviewer for the suggestion to add more detail of the results from the manuscript to be added to the abstract. Further detail, including the resiliency measures identified and their psychometric properties, has been added into the results section of the abstract. | Abstract  **Background:** People with brain injury can have lower resiliency compared to the general public. Yet, resiliency can facilitate positive processes to negotiate adversity after brain injury. It is therefore important to measure resiliency after a brain injury. **Objective:** The review aimed (1) to identify self-report resiliency outcome measures for use with people after acquired brain injury, using the process-based Traumatic Brain Injury Resiliency Model as the guiding conceptual framework, and (2) to summarize the psychometric properties of the identified outcome measures and the utility of these measures in clinical rehabilitation. **Method:** The COSMIN guidelines for systematic reviews were followed to ensure appropriate reporting for each measure. Databases CINAHL, EMBASE, Medline, and PsychINFO were searched and independently reviewed by two people. |
| In the conclusion, the authors discuss three resilience outcomes delineated by the Traumatic Brain Injury Resiliency Model. It is recommended to incorporate these three outcomes into the methodological guidelines mentioned in the study. | We have now included more explanation of the resiliency-related outcomes in the methods section. We have also aligned our results section to the three resiliency-related outcomes. | Page 5, paragraph 4:  Specifically, the TBIRM highlights three key resiliency-related outcomes including: Re-engaging in Activities (e.g., participation in normal activities), Adjusting Participation Patterns and Preferences (e.g., accept and adapt), and Reconstructing Identity (e.g., accepting disability and creating new goals) [11].  Page 9, paragraph 2:  To understand how each measure aligned with the TBIRM, two authors (JG and JS) independently reviewed the purpose of the measure and individual items of each measure. Authors then independently identified which of the three resiliency-related outcomes of the TBIRM the measure was most aligned (e.g., Reconstructing Identity, Re-engaging in Activities, and Adjusting Participation Patterns and Preferences). |
| To enhance clarity, the results section could be divided into two subsections, each accompanied by relevant tables.  The first subsection could outline the general characteristics of the included studies, such as country of origin, sampling settings, participant demographics, and details of the questionnaires utilized. The second subsection could focus on presenting the identified outcomes, including the questionnaires employed, their dimensions and sub-dimensions, the number of items within each, and the overall number of items per questionnaire. Psychometric properties should be presented alongside these findings. | We thank the reviewer for the suggestion and agree that the presentation of results can be improved. We have now modified the tables to better showcase the results. Table 2 describes the psychometric properties of each assessment included in the review. Table 3 and corresponding second section focuses on the TBIRM and where each measure falls within the subsections of Reconstructing Identity, Re-engaging in Activities, and Adjusting Participation Patterns and Preferences. | Page 11 – 21, Table 2. Self-Report Resiliency Measures Summary and Psychometric Properties  Page 22, Table 3. Checklist of Constructs Assessed by Measures |
