# Supplementary material for: An exploratory review of resiliency assessments after brain injury
Source: PLoS One. 2025 Jan 3;20(1):e0292502. doi: 10.1371/journal.pone.0292502 (PMC11698413; doi:10.1371/journal.pone.0292502)
Supplement: Attachment — Submitted filename: Response to Reviewers_4.docx [file pone.0292502.s006.docx]

**Response to Reviewer 3**

**Introduction**

| **Reviewer Comment** | **Response to Reviewer** | **Location in Manuscript** |
| --- | --- | --- |
| Paragraph 1 sentence 3: “greater impairment” is vague and would benefit from clarity. | We have now included more description to address reviewer comments. | Page 4, Line 45-47:  “For example, the presence of depression during rehabilitation has been associated with decreased health-related quality of life at long-term follow-up.” |
| Sentence 4: specify the direction of quality of life changes. | We have now specified the direction of the quality of life changes and added more context. | Page 4, Line 47-48  “Long-term, consequences of living with brain injury can result in unemployment and decreased quality of life.” |
| Given resiliency is the key construct, an explicit definition is important to include at some early point in the introduction. This is included at the start of the third paragraph but is warranted earlier on, prior to any discussion of this construct. | Thank you for this insight. Paragraph 3 has now been moved to be paragraph 2, providing a definition of resiliency earlier in the introduction to increase clarity. |  |
| Paragraph 2: Ensure you have defined the acronym “TBI”. | The acronym has now been defined. | Page 5, Line 63 |
| Paragraph 2 final sentence: please add a reference. | The sentence has been taken out as it was deemed redundant with the previous sentence. |  |
| Paragraph 3 final sentence: “resiliency relates to the many factors contributing to the process of resiliency” is unclear. This definition is circular, in that resiliency is described as contributing to resiliency. Re-wording this for clarity if recommended. | To increase clarity, the definition has now been clarified. | Page 4, Line 60-61  “According to the TBIRM, resilience encompasses an individual’s trait or state, while resiliency relates to an individual’s process and the many factors it influences[11].” |
| You would benefit from a clearer rationale leading up the aims of this study, to more directly highlight why measures need to be identified and reviewed. It is noted that brain injury rehabilitation does not commonly address resiliency. This could be due to several factors, such as higher importance placed on other domains, lack of time, and/or lack of standardised procedures and tools. Your introduction has well established the importance of resiliency and enhancing this – what does the role of a measure play? What are the current gaps in our knowledge and use of measures? Your study identified 9 measures that I assume have been used within research and perhaps clinical settings, therefore more explicit rationale around why this review is necessary is needed. | We have now added more modified the sentences to highlight the rationale for the review. These sentences provide the knowledge that previous work (the TBIRM) has introduced the concept of resiliency, and this review attempts to identify measures to put those concepts into practice. | Line 70-71  “The process of resiliency in brain injury, introduced by the TBIRM, is a new concept with promising utility.”  Line 75-77  “The identification of measures assessing resiliency in brain injury populations using the TBIRM conceptual framework can facilitate use of these measures in clinical practice and research.” |

**Method**

| **Reviewer Comment** | **Response to Reviewer** | **Location in Manuscript** |
| --- | --- | --- |
| “Measures that were used on acquired brain injury population (e.g., TBI stroke) were included given similar experiences with disability after injury”. This is unclear, as based on the introduction my understanding was that the study was focussing on brain injury. Please clarify, perhaps within your aims, whether brain injury or broader disability was the focus. | We have now re-configured the inclusion criteria to state participant inclusion criteria at the beginning, with population examples stated in brackets. | Page 8, line 148-150  “Article inclusion criteria were as follows: (a) the clinical measure assessed resiliency according to the TBIRM in adults with acquired brain injuries (e.g., TBI, stroke)…” |

**Results**

| **Reviewer Comment** | **Response to Reviewer** | **Location in Manuscript** |
| --- | --- | --- |
| “The measures are reasonable in length for ease of use clinically, in a range from 10-35 statements”. What is the basis for this claim? It is unclear whether this is evidence-based or the authors’ interpretation of “reasonable in length”. | To increase clarity, the sentence has been changed. | Line 196  “The measures range from 10-35 statements.” |
| The results section reads well, however can be presented in a briefer format. At present, there is a lot of detail describing each scale and this in some cases duplicates information presented in Table 2, such as example items and the population in which the scale has been validated. Text and tables should complement rather than duplicate the other. I feel the lengthy description of each scale is not needed in the latter part of the results section; this could focus more on how each scale maps onto the construct. | We appreciate the comment, we have deleted all repetitive examples already stated in Table 2. |  |

**Discussion**

| **Reviewer Comment** | **Response to Reviewer** | | **Location in Manuscript** |
| --- | --- | --- | --- |
| Line 306: “While some important factors and experiences overlap among subpopulations within brain injury (e.g., stroke versus traumatic brain injury)”. This claim would benefit from specific examples of factors. | | We appreciate the comment and have now changed the paragraph and refocused our commentary, and have deleted the sentence. | Line 335-340  “There was variability in the validation of measures to adult brain injury sub-populations (e.g., stroke, TBI) and time since injury… Future research could consider further developing psychometrics of measures for all brain injury population groups.” |
| As per my earlier comment on the introduction, your discussion would benefit from more explicit note around why a valid tool is needed that addresses all aspects of resiliency. What role does a measure play in clinical settings and why is this needed? | | We have now included a section to more fully address clinical relevance, describing the use and benefit of a resiliency measure in clinical practice. | Line 330-335  “When considering clinical relevance, some measures included in our review may have more clinical utility. Similar to selecting measures for other constructs, clinicians may consider person-level aspects of the measure (e.g., population for which the measure has been validated and the stage of the person’s recovery) as well as the accessibility of measures in clinical practice (e.g., cost of the measure, training required). For resiliency measures in particular, clinicians may also want to consider how the measure aligns with specific constructs within the TBIRM framework, as presented in our review. In this way, future research can identify potential outcome measures for use alongside exploration of the processes of developing resiliency after brain injury.” |

**Limitations**

| **Reviewer Comment** | **Response to Reviewer** | **Location in Manuscript** |
| --- | --- | --- |
| Make sure there is evidence to support your claims. It is important to not just list limitations, but also explain how. “…as resiliency could vary across languages and non-English speaking regions” – is there evidence to support that resiliency might have cultural/language differences? | We appreciate the reviewer comments and have now made changes to the sentence to indicate that the understanding of resiliency may vary, as well as added a supporting reference. | Line 378, Reference 45  “…as the understanding of the concept of resiliency could vary across languages and cultures [45].” |
