# Supplementary material for: An exploratory review of resiliency assessments after brain injury
Source: PLoS One. 2025 Jan 3;20(1):e0292502. doi: 10.1371/journal.pone.0292502 (PMC11698413; doi:10.1371/journal.pone.0292502)
Supplement: Attachment — Submitted filename: Response to Reviewers.docx [file pone.0292502.s007.docx]

**Response to Reviewers**

The revised manuscript attached has been changed to reflect the journal reference requirements.
